# Entropy optimization and heat transfer analysis in MHD Williamson nanofluid flow over a vertical Riga plate with nonlinear thermal radiation

**DOI:** 10.1038/s41598-021-97874-4

**Published:** 2021-09-15

**Authors:** Muhammad Rooman, Muhammad Asif Jan, Zahir Shah, Poom Kumam, Ahmed Alshehri

**Affiliations:** 1 Institute of Numerical Sciences, Kohat University of Science and Technology KUST, Kohat, 26000 Khyber Pakhtoonkhwa Pakistan; 2 Department of Mathematical Sciences, University of Lakki Marwat, Lakki Marwat, 28420 Khyber Pakhtunkhwa Pakistan; 3 Fixed Point Research Laboratory, Fixed Point Theory and Applications Research Group, Center of Excellence in Theoretical and Computational Science (TaCS-CoE), Faculty of Science, King Mongkut’s University of Technology Thonburi (KMUTT), 126 Pracha Uthit Rd., Bang Mod, Thung Khru, Bangkok, 10140 Thailand; 4 Department of Medical Research, China Medical University Hospital, China Medical University, Taichung, 40402 Taiwan; 5 Department of Mathematics, Faculty of Sciences, King Abdulaziz University, Jeddah, 21589 Saudi Arabia

**Keywords:** Engineering, Mathematics and computing, Physics

## Abstract

The entropy generation for a reactive Williamson nanofluid flow past a vertical Riga system is the subject of this article. The effects of MHD, thermophoresis, nonlinear heat radiation and varying heat conductivity are modeled into the heat equation in the established model. Suitable similarity transformations are examined to bring down the partial differential equations into ordinary differential equations. The Homotopy analysis approach is used to solve the dimensionless transport equations analytically. The graphic information of the various parameters that emerged from the model is effectively collected and deliberated. The temperature field expands with thermophoresis, Brownian motion and temperature ratio parameters as the modified Hartmann number forces an increase in velocity, according to the findings of this analysis. With the increase in the fluid material terms, the entropy generation and Bejan number increase. Riga plate has numerous applications in improving the thermo-physics features of a fluid, the value of magnetic field embraces an important role in fluid mechanics. An external electric field can be used to control flow in weak electrically conductive fluids. The Riga plate is one of the devices used in this regard. It’s a device that creates electromagnetic fields. They produce the Lorentz force which is a force that directs fluid flow. The authors have discussed the entropy optimization for a reactive Williamson nanofluid flow past a vertical Riga plate is addressed. This is the first investigation on mass and heat transfer flow that the authors are aware of, and no similar work has yet been published in the literature. A thorough mathematical examination is also required to demonstrate the model’s regularity. The authors believe that the results acquired are novel and have not been plagiarized from any other sources.

## Introduction

Modern research has discovered that high-profile freezing is compulsory for the majority of industrial and technological operations. By deprived thermo-physical properties of the typical fluid, the extraordinary heat mass flux efficiency is difficult to achieve. The issue was talked to a certain amount by the overview of the notion of nanoliquids. A nanoliquid is a liquid drenched by extremely conductive metallic particles of Nano-size. As compared to larger particles, nanoparticles will easily remain suspended in the base liquid for an extended period of time. Following Choi^1^ research and corresponding reviews, nanofluids appear to be organizations encouraged in mass and heat transfer applications, specifically in heat exchange, aerospace technology, micro processing, refrigeration and automotive, etc. in which extremely energetic products are elaborated with both a slight size and compressed form. Nanoliquids are also significant from a medical standpoint. The applications of nanofluids in medical field are the treatment of hyperthermia, therapy for cancer care, surgeries, openings and wounds/blocking the veins etc. Buongiorno^2^ presented the knowledge of nanoliquid and established a mathematical model to analyze and explore the thermal features of base fluids. Later on, various expansions have been made in the nanofluids field. Mustafa et al.^3^ founded an analytical solution of nanofluid flow adjacent to a stagnation point near a stretching shallow by using HAM. Turkyilmazoglu^4^ founded exact analytical solution for MHD nanofluid flow for transfer of mass and heat across a porous shrinking surface. Nield and Kuznetsov^5^ presented an analytical solution for the problem of Cheng–Minkowycz in a medium with pores drenched by nanoliquid. The energy conservation problems of convection conjugate conduction and heat transfer radiation with magnetic effects and viscous dissipation were investigated by Hsiao^6^. Theoretically laminar flow and rate of hear transfer of water/alumina nanoparticles favored a rounded microchannel in the incidence of the uniform magnetic field were investigated by Malvandi and Ganji^7^.

Because of its numerous applications in improving the thermo-physics features of a fluid, the value of the magnetic field embraces an important role in fluid mechanics. Various liquids that are weak conductors of electricity are encountered in fields for instance earth sciences and astrology. As a result, an external agent is often required to improve phenomena of heat flow via improved conductivity and other thermo-physics properties. A magnetic piece or a forever fixed series of these magnets with discontinuous electrodes could be used as this external agent. Gailitis and Lielausis^8^ was the first to use this type of formulation in Riga and it was formally introduced Riga Plate. The Riga plate as constructed is much useful because it has rapidly become well-known in industrial procedures causing fluid flow behavior. Ahmed et al.^9^ investigated the effects of surface heating through no mass flux. The effect of the Lorentz force, which decays significantly with growing movement from the plate’s surface, was used in the model by using the Riga plate. Sheikholeslami et al.^10^ investigated the effect of Lorentz forces on nanofluid Marangoni convection. Shafiq et al.^11^ investigated the flow of fluid points across a Riga plate using the Walters-B model. Adeel et al.^12^ has examined the mixed convection of nanoparticles in a fluid flow with a vertically mounted Riga plate. Thermal radiation’s effect on Marangoni convective nanoliquid flow against a Riga plate was studied by Rasool et al.^13^.

The most prevalent non-Newtonian fluids observed are pseudoplastic fluids. Because of its broad collection of applications in industry, polymers with a high molecular weight melt, emulsion of coated sheets such as photographic films, polymer layer extrusion, and so on are examples; the analysis of the pseudoplastic fluid flow at the boundary layer is of keen importance. The Navier Stokes equations by themselves are insufficient to describe fluid rheological characteristics. As a consequence, rheological models have been suggested to resolve this shortcoming. Many models are being suggested to describe the Structure of pseudoplastic fluids, including the Carreau model, power-law model, Ellis fluid model, and Cross model, but Williamson fluid model has received slight attention.

The Williamson fluid is one the most essential non-Newtonian fluids having reduced viscosity as shear stress rises and features that are quite comparable to polymeric solutions. In other words, in the Williamson fluid, the functional viscosity should decrease forever as the shear rate rises with infinite viscosity at rest and nil viscosity as the shear rate approaches infinity. Williamson^14^ studied the pseudoplastic flow substances and formulated a model equation to explain the pseudoplastic fluids flow, which he then tested experimentally. The movement of a tinny film of a Williamson fluid by an inclined surface by means of a gravitational field was investigated by Lyubimov and Perminov^15^. Williamson fluid-applied into a rock fragment perturbation solution was investigated by Dapra and Scarpi^16^. Nadeem et al.^17^ addressed the Williamson fluid of peristaltic flow. Vasudev et al.^18^ investigated the heat transfer effects of peristaltic thrusting of a Williamson fluid over a medium with pores. Nadeem et al.^19^ examined the Williamson fluid model’s two-dimensional flow through a stretching sheet. Compared to other versions, this one best suits the investigational statistics polymer solutions and particle interruptions, according to Cramer et al.^20^. The power law theory shows that when the shear rate reaches infinity, the apparent/effective viscosity should decrease forever, resulting in at rest, the viscosity is infinite and as the shear rate increases to infinity, the viscosity is zero. A natural fluid has both higher and lower active viscosity based on the molecular nature of the fluid. Both the maximum and minimum are put into consideration in the Williamson fluid model. As a result, it will produce better results for pseudoplastic fluids. Ahmad et al.^21^ explored the Maxwell nanofluid transfer between two coaxially simultaneous stretchy rotating disks in the context of an axial magnetic field and varying thermal conductivity. They applied the Buongiorno nanofluid model to demonstrate upper and lower disks behavior in both the same and opposing orientations. Turkyilmazoglu^22^ investigated the influence of magnetic fields and slip on the flow and heat transfer of a stagnation point Jeffrey fluid on deformed objects. Abo-Elkhair et al.^23^ established the modest Reynolds number and imposed magnetic field impacts on the hybrid Bio-nanofluid via a peristaltic channel in the dispersion of nanoparticles such as gold and copper. Bhatti et al.^24^ examined the applicability of the slip occurrence in bioconvection characteristics in a non-Newtonian Eyring–Powell nanofluid model constrained by a stretching sheet. References list a number of useful and fruitful papers on nanofluid research and various approaches^25–31^.

Motivated from the above stated facts is to examine entropy optimization and heat transfer analysis in MHD Williamson nanofluid over a vertical Riga plate with nonlinear thermal radiation. The novel aspect of the current study is to determine irreversibility for reactive Williamson nanofluid transport over a Riga vertical porous system with nonlinear convection. The study was prompted by a number of presentations on Williamson nanoliquid flow and its relevant characteristics in engineering and manufacturing. The thermodynamic second law considers complete entropy optimization for irreversibility. The problem is explained analytically using the HAM. Different flow characteristics are investigated, and the results are graphed.

## Mathematical formulation

**Figure 1:**
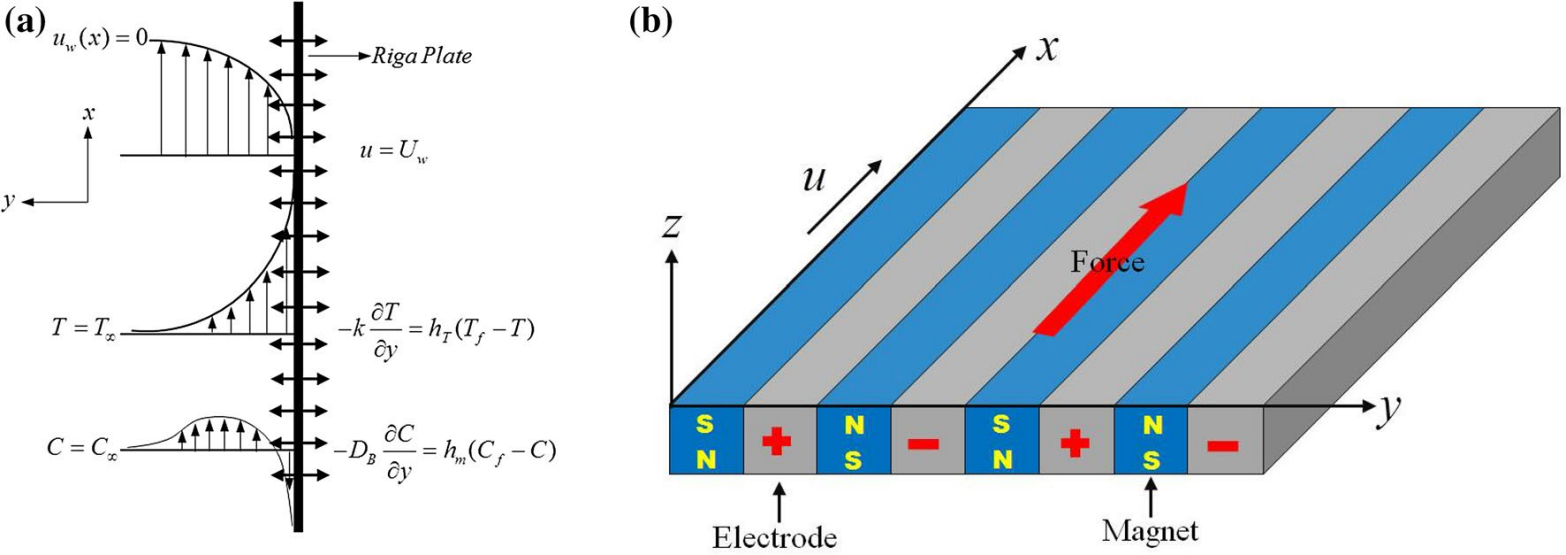
(**a**) The fluid flow geometry. (**b**) Lorentz force induced Riga plate.

Consider a vertical Riga plate with a two-dimensional, steady, incompressible and reactive Williamson nanofluid flow. The plate’s coordinates $(x,y)$ corresponds to the velocity components $(u,v)$ see (Fig. 1a). As shown in Fig. 1a,b the x-axis is aligned with the flow direction, whereas the y-axis is parallel to it. The Riga plate is built up of different arrays that are split into electrodes and magnets which are mounted on the board, the width of electrodes and magnets is denoted by $s$. The surface stretches non-linearly velocity $u={U}_{w}=b{x}^{n}$ where $b$ represents the stretching rate, ${U}_{w}$ represents the surface velocity and $n$ is a power index. The Lorentz force is generated when the Riga plate’s electromagnetic field interacts with the nanoparticles contained in the base fluid.

Magnetic hydrodynamics (MHD) is used in this issue (Fig. 1b), and the effect of a higher-order chemical reaction is factored into the nanomaterial concentration equation. Meanwhile, the energy equation investigates the occurrence of nonlinear thermal radiation, thermophoresis, and Brownian diffusion. Surface mass flux, convective thermal and mass boundary conditions also have an effect on the model.

The Cauchy stress tensor ${\varvec{S}}$ for Wiliamson fluid model is^17^

1 $${\varvec{S}}=-p{\varvec{I}}+{\varvec{\tau}}$$

The concept of extra Stress ${\varvec{\tau}}$ for Wiliamson fluid is

2 $${\varvec{\tau}}=\left[{\mu }_{\infty }+\frac{\left({\mu }_{0}-{\mu }_{\infty }\right)}{1-\boldsymbol{\Gamma }\dot{\gamma }}\right]{{\varvec{A}}}_{1}$$

where ${\mu }_{0}$ represents limiting viscosity at zero shear rate, ${\mu }_{\infty }$ represents limiting viscosity at an infinite shear rate, ${{\varvec{A}}}_{1}$ represents the first Rivlin–Erickson tensor, $\boldsymbol{\Gamma }>0$ is time constant and $\dot{\gamma }$ is defined as

3 $$\dot{\gamma }=\sqrt{\frac{1}{2}trace\left({{{\varvec{A}}}_{1}}^{2}\right)}$$

Here we assumed the case for which ${\mu }_{\infty }=0$ and $\boldsymbol{\Gamma }\dot{\gamma }<1$, so by using binomial expansion Eq. (3) can be written as:

4 $${\varvec{\tau}}={\mu }_{0}\left[1+\boldsymbol{\Gamma }\dot{\gamma }\right]{{\varvec{A}}}_{1}$$

Modeling temperature-dependent thermal conductivity is expressed as

5 $$k\left(T\right)={k}_{\infty }\left(1+\delta \frac{T-{T}_{\infty }}{{T}_{f}-{T}_{\infty }}\right)$$

where ${k}_{\infty }$ stands for thermal conductivity at room temperature and $\delta$ stands for its parameter. The derived transport equations are expressed as with the highlighted assumptions and Oberbeck–Boussinesq associated with the well-known boundary layer approximation.

6 $$\frac{\partial u}{ \partial x}+\frac{\partial v}{\partial y}=0$$

7 $$\begin{gathered} u\frac{\partial u}{{\partial x}} + v\frac{\partial u}{{\partial y}} = \nu \frac{{\partial^{2} u}}{{\partial y^{2} }} + \sqrt 2 \nu{\varvec{\varGamma}}\frac{\partial u}{{\partial y}}\frac{{\partial^{2} u}}{{\partial y^{2} }} + \frac{{\pi j_{0} M^{*} exp\left( { - \frac{\pi y}{s}} \right)}}{{8\rho_{f} }} - \frac{{\sigma B_{0}^{2} u}}{\rho } \hfill \\ + \frac{1}{{\rho_{f} }}\left[ {\left( {1 - C_{\infty } } \right)\rho_{f} \left( {T - T_{\infty } } \right)\beta_{T} - \left( {\rho_{p} - \rho_{f} } \right)\left( {C - C_{\infty } } \right)} \right]g \hfill \\ \end{gathered}$$

8 $${\left(\rho {c}_{p}\right)}_{f}\left(u\frac{\partial T}{\partial x}+v\frac{\partial T}{\partial y}\right)=\frac{\partial }{\partial y}\left(k\left(T\right)\frac{\partial T}{\partial y}\right)+{\left(\rho {c}_{p}\right)}_{p}\left[\frac{{D}_{T}}{{T}_{\infty }}{\left(\frac{\partial T}{\partial y}\right)}^{2}+{D}_{B}\left(\frac{\partial T}{\partial y}\frac{\partial C}{\partial y}\right)\right]+\frac{16{a}^{*}}{3{b}^{*}}\frac{\partial }{\partial y}\left({T}^{3}\frac{\partial T}{\partial y}\right)$$

9 $$u\frac{\partial \phi }{\partial x}+v\frac{\partial \phi }{\partial y}={D}_{B}\frac{{\partial }^{2}C }{\partial {y}^{2}}+\frac{{D}_{T}}{{T}_{\infty }}\left(\frac{{\partial }^{2}T}{\partial {y}^{2}}\right)-{k}_{1}\left(C-{C}_{\infty }\right)$$

The Boundary Conditions are listed as:

$$u={U}_{w}=b{x}^{n}, v={V}_{w}, -k\frac{\partial T}{\partial y}={h}_{1}\left({T}_{f}-T\right), -{D}_{B}\frac{\partial C}{\partial y}={h}_{2}\left({C}_{f}-C\right) at y=0$$

10 $$u\to 0, T\to {T}_{\infty }, C\to {C}_{\infty }, as y\to \infty$$

The convective surface concentration and temperature are represented by ${C}_{f}$ and ${T}_{f}$ respectively, ${h}_{1}=a{x}^{(n-1)/2}, {h}_{2}=c{x}^{(n-1)/2}$ depicts the coefficient of heat and mass transfer respectively, the term ${V}_{w}={V}_{0}{x}^{(n-1)/2}$ describes the suction/injection, where $a, c$ and ${V}_{0}$ are constant. Various symbols used in this research are given in Table.1.

**Table 1:** Various symbols appear in the governing equations.

Symbols	Description	Symbols	Description
*u,v*	Velocity in *x,y* direction	*k*	Thermal conductivity
*ν*_*f*_	Base fluid kinematic viscosity	*C*	Nanoparticle concentration
*ρ*_*f*_	Base fluid density	*F*_1_	Nan-uniform inertia coefficient
*μ*	Fluid viscosity	*U*_*w*_	Velocity at the sheet
*We*	Fluid material constant	*g*	Acceleration due to gravity
*T*	Temperature	M*	magnetic effect
*T*_*∞*_	Temperature at free stream	*β*_*T*_	Coefficient of thermal diffusion
*c*_*p*_	Specific heat	*C*_*∞*_	Free stream nanoparticles concentration
*n*	Power index	*k*_1_	Chemical reaction rate
*D*_*B*_	Brownian diffusion coefficient	*D*_*T*_	Thermophoretic diffusion coefficient
*ρ*_*p*_	Nanoparticles density	(*ρc*_*p*_)_*f*_	Base fluid heat capacity
(*ρc*_*p*_)_*p*_	Nanoparticles heat capacity	*j*_0_	Current density
*ρ*	Fluid density	*σ*	Electrical conductivity
*B*_0_	Uniform magnetic field intensity	*s*	Breadth of magnets and electrodes

The dimensionless variables mentioned below are inserted into the main equations.

11 $$\eta =\sqrt{\frac{b\left(n+1\right){x}^{n-1}}{2\nu }}y, \psi =\sqrt{\frac{2\nu b{x}^{n+1}}{n+1}}f\left(\eta \right), \theta \left(\eta \right)=\frac{T-{T}_{\infty }}{{T}_{f}-{T}_{\infty }}, h\left(\eta \right)=\frac{C-{C}_{\infty }}{{C}_{f}-{C}_{\infty }}, u=\frac{\partial \psi }{\partial y}, v=-\frac{\partial \psi }{\partial x}$$

The continuity Eq. (6) becomes true when the quantities in Eq. (11) are substituted into the governing Eqs. (6–9), while Eqs. (7–9) yield the under listed:

12 $${f}^{\prime\prime\prime}+We{f}^{\prime\prime}{f}^{\prime\prime\prime}-\frac{2n}{n+1}{{f}^{\prime}}^{2}+f{f}^{\prime\prime}-\frac{2}{n+1}{M}_{0}{f}^{\prime}+\frac{2}{n+1}H{e}^{\left(-B\eta \right)}+\frac{2}{n+1}{\lambda }_{1}\left(\theta -Rh\right)=0$$

13 $$\frac{1}{{P}_{r}}\left[1+\delta \theta +{N}_{r}{\left(1+\left({\theta }_{b}-1\right)\theta \right)}^{3}\right]{\theta }^{\prime\prime}+\frac{3}{{P}_{r}}\left[{N}_{r}\left({\theta }_{b}-1\right){\left(1+\left({\theta }_{b}-1\right)\theta \right)}^{2}\right]{{\theta }^{\prime}}^{2}+\delta {{\theta }^{\prime}}^{2}+f{\theta }^{\prime}+{N}_{t}{{\theta }^{\prime}}^{2}+{N}_{b}{\theta }^{\prime}{h}^{\prime}=0$$

14 $${h}^{\prime\prime}+Sc\left(f{h}^{\prime}-\frac{2}{n+1}{\gamma }_{1}h\right)+\frac{{N}_{t}}{{N}_{b}}{\theta }^{\prime\prime}=0$$

The dimensionless boundary conditions are

15 $${f}^{\prime}\left(0\right)=1, f\left(0\right)={F}_{w}, {\theta }^{\prime}\left(0\right)=-{B}_{1}\left(1-\theta \left(0\right)\right), {h}^{\prime}\left(0\right)=-{B}_{2}\left(1-h\left(0\right)\right), {f}^{\prime}\left(\infty \right)=0, \theta \left(\infty \right)=0, h\left(\infty \right)=0$$

### The entropy generation equation

The volumetric rate of entropy generation for the Williamson fluid influenced by nonlinear thermal radiation and diffusion effect is modeled using thermodynamics second law

16 $${S}_{Gn}={S}_{ht}+{S}_{d}$$

where ${S}_{Gn}$ denotes volumetric entropy generation, ${S}_{ht}$ denotes entropy generation of heat transfer or conduction and ${S}_{d}$ denotes entropy generation of mass transfer effects over a finite temperature and concentration difference. As a result, ${S}_{Gn}$ can be written as:

17 $${S}_{Gn}=\frac{1}{{T}^{2}}\left(k+\frac{16{a}^{*}{T}^{3}}{3{b}^{*}k}\right){\left(\frac{\partial T}{\partial y}\right)}^{2}+\left[\frac{R{D}_{B}}{C}{\left(\frac{\partial C}{\partial y}\right)}^{2}+\frac{R{D}_{B}}{T}\left(\frac{\partial C}{\partial y}\frac{\partial T}{\partial y}\right)\right]$$

For the applied boundary conditions (10), the characteristic entropy generation ${S}_{G}^{\prime\prime\prime}$ is given as

18 $${S}_{G}^{\prime\prime\prime}={k}_{\infty }\frac{{\left({T}_{w}-{T}_{\infty }\right)}^{2}\left(n+1\right)}{2{T}_{\infty }^{2}{x}^{2}}$$

The generation of dimensionless entropy can be written as

19 $${N}_{Gs}=\frac{Re\left[1+\delta \theta +{N}_{r}{\left(1+\left({\theta }_{b}-1\right)\theta \right)}^{3}\right]}{{\left(1+\left({\theta }_{b}-1\right)\theta \right)}^{2}}{{\theta }^{\prime}}^{2}+\frac{Re{\gamma }_{2}\left({C}_{b}-1\right)}{\left({\theta }_{b}-1\right)}\left[\frac{\left({C}_{b}-1\right){{C}^{\prime}}^{2}}{\left(1+\left({C}_{b}-1\right)C\right)\left({\theta }_{b}-1\right)}+\frac{\theta {^{\prime}}C{^{\prime}}}{1+\left({\theta }_{b}-1\right)\theta }\right]=0$$

The entropy output number is represented by the word ${N}_{Gs}=\frac{{S}_{Gn}}{{S}_{G}^{\prime\prime\prime}}={N}_{h}+{N}_{m}$. ${N}_{h}$ is the first term in the RHS of Eq. (19), which denotes entropy generation caused by heat transfer, while ${N}_{m}$ denotes entropy generation caused by mass transfer.

### Bejan number

The entropy generation number ${N}_{Gs}$ determines the distribution of entropy generation in the flow area, but in an energy optimization problem, the contribution of thermal conductivity to total entropy output is needed. The Bejan number (Be) expresses the value of thermal irreversibility in comparison to absolute irreversibility and is used to describe a number.

20 $$Be=\frac{{N}_{h}}{{N}_{Gs}}$$

The emerging parameters from the governing equations are described as:

21 $$\begin{aligned} \theta_{b} & = \frac{{T_{f} }}{{T_{\infty } }}, We = \frac{\Gamma }{2}\sqrt {\frac{{b^{3} \left( {n + 1} \right)x^{3n - 1} }}{\nu }} , M_{0} = \frac{{\sigma B_{0}^{2} x}}{b\rho }, H = \frac{{\pi j_{0} M^{*} }}{{8\rho_{f} b^{2} x^{2n - 1} }}, \\ B & = \frac{\pi }{s}\sqrt {\frac{2\nu }{{b\left( {n + 1} \right)x^{n + 1} }}} ,\lambda_{1} = \frac{{g\beta_{T} \left( {1 - C_{\infty } } \right)\rho_{f} \left( {T_{f} - T_{\infty } } \right)}}{{b^{2} \rho_{f} x^{2n - 1} }}, R = \frac{{\left( {\rho_{p} - \rho_{\infty } } \right)\left( {C_{f} - C_{\infty } } \right)}}{{\beta_{T} \left( {1 - C_{\infty } } \right)\left( {T_{f} - T_{\infty } } \right)\rho_{f} }}, \\ P_{r} & = \frac{{\mu_{f} c_{p} }}{{k_{\infty } }} , N_{r} = \frac{{16a^{*} T_{\infty }^{3} }}{{3b^{*} k_{\infty } }} N_{b} = \frac{{\left( {\rho cp} \right)_{p} D_{B} \left( {C_{\omega } - C_{\infty } } \right)}}{{\nu_{f} \left( {\rho cp} \right)_{f} }}, \\ Re & = \frac{{bx^{n - 1} }}{{\nu_{f} }}, B_{1} = \frac{a}{{k_{\infty } }}\sqrt {\frac{2\nu }{{b\left( {n + 1} \right)}}} , B_{2} = \frac{c}{{D_{B} }}\sqrt {\frac{2\nu }{{b\left( {n + 1} \right)}}} , N_{t} = \frac{{\left( {\rho cp} \right)_{p} D_{T} \left( {T_{\omega } - T_{\infty } } \right)}}{{\nu_{f} \left( {\rho cp} \right)_{f} }} \\ \end{aligned}$$

The modeled parameter after simplification are given in Table 2.

**Table 2:** lists of the evolving physical quantities that join the dimensionless governing equations.

Parameter	Description	Parameter	Description
*We*	Material parameter	*λ*_1_	Mixed convection term
*θ*_*b*_	Temperature ratio term	*R*	Buoyancy ratio term
*N*_*r*_	Radiation term	*θ*_*r*_	Wall temperature excess ratio
*γ*_1_	Chemical reaction	*P*_*r*_	Prandtl number
*γ*_2_	Diffusion Constant	*H*	Modified Hartmann number
*N*_*t*_	Thermophoresis parameter	*δ*	Thermal conductivity term
*B*_1_	Thermal Biot number	*Da*	Darcy number
*B*_2_	Mass Biot number	*Sc*	Schmidt number
*B*_*r*_	Brikman number	*N*_*b*_	Brownian motion parameter

Engineers are particularly interested in the skin friction coefficient ${c}_{f}$, the local Nusselt number ${N}_{u}$ and the local Sherwood number ${S}_{h}$, which are all ordered as

22 $${c}_{f}=\frac{{\tau }_{\omega }}{\rho {U}_{\omega }^{2}}, {N}_{u}=\frac{x{q}_{\omega }}{k\left({T}_{f}-{T}_{\infty }\right)}, {S}_{h}=\frac{x{q}_{m}}{{D}_{B}\left({C}_{f}-{C}_{\infty }\right)}$$

where ${\tau }_{\omega }, {q}_{\omega }$ and ${q}_{m}$ are defines the shear stress, heat flux and mass flux at the surface respectively. These are expressed as

23 $${\tau }_{\omega }={\left.\nu \left(\frac{\partial u}{\partial y}+\frac{1}{2}\nu \Gamma {\left(\frac{\partial u}{\partial y}\right)}^{2}\right)\right|}_{y=0}, {q}_{m}{\left.-\left(k+\frac{16{T}^{3}{a}^{*}}{3k{b}^{*}}\right)\frac{\partial T}{\partial y}\right|}_{y=0}, {q}_{m}={\left.-{D}_{B}\left(\frac{\partial C}{\partial y}\right)\right|}_{y=0}$$

In view of Eqs. (11) and (23), the quantities in (22) respectively yields (24–26)

24 $${\tau }_{\omega }={\left(\frac{n+1}{2}\right)}^{1/2}\left({f}^{\prime\prime}\left(0\right)+\frac{We}{2}{\left({f}^{\prime\prime}\left(0\right)\right)}^{2}\right){Re}^{-1/2}$$

25 $${N}_{u}=-{\left(\frac{n+1}{2}\right)}^{1/2}\left(1+{N}_{r}{\left(1+\left({\theta }_{r}-1\right)\theta \left(0\right)\right)}^{3}\right){Re}^{1/2}{\theta }^{\prime}\left(0\right)$$

26 $$S_{h} = - \left( {\frac{n + 1}{2}} \right)^{1/2} Re^{1/2} h^{\prime } \left( 0 \right)$$

## Result and discussion

### Velocity profile

**Figure 2:**
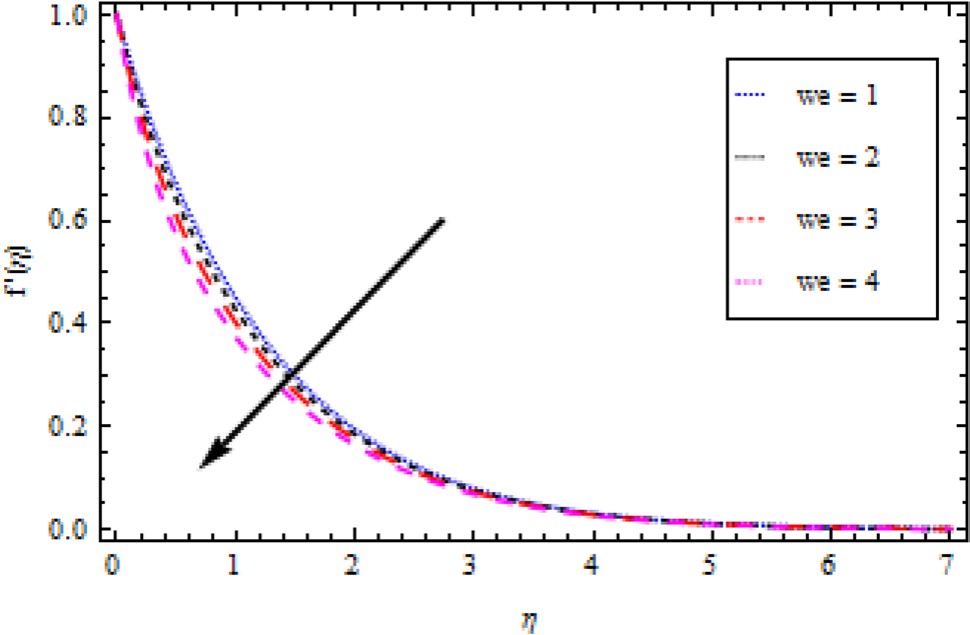
Velocity profile variation with respect to *We*.

**Figure 3:**
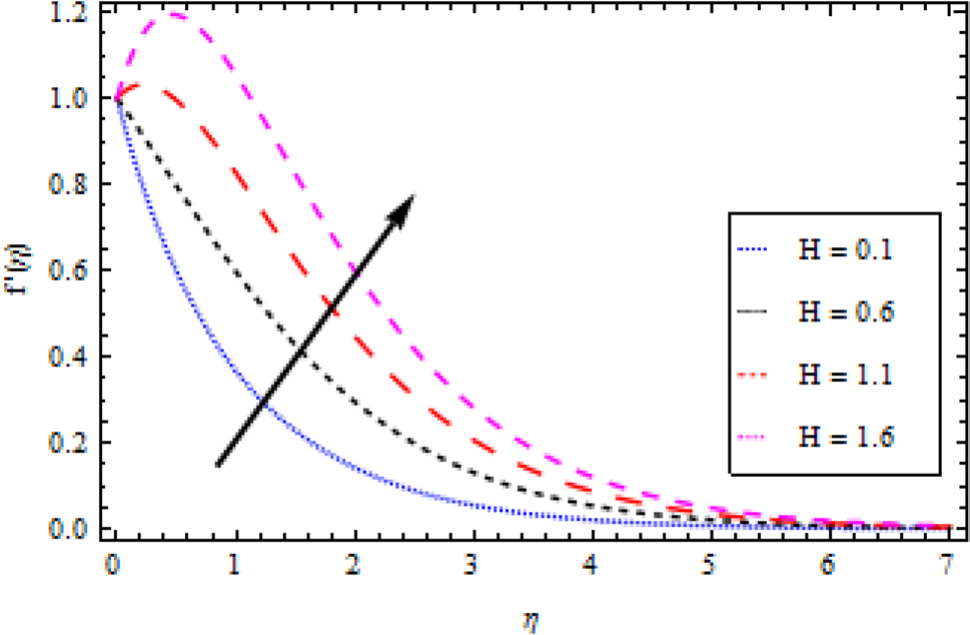
Velocity profile variation with respect to *H*.

**Figure 4:**
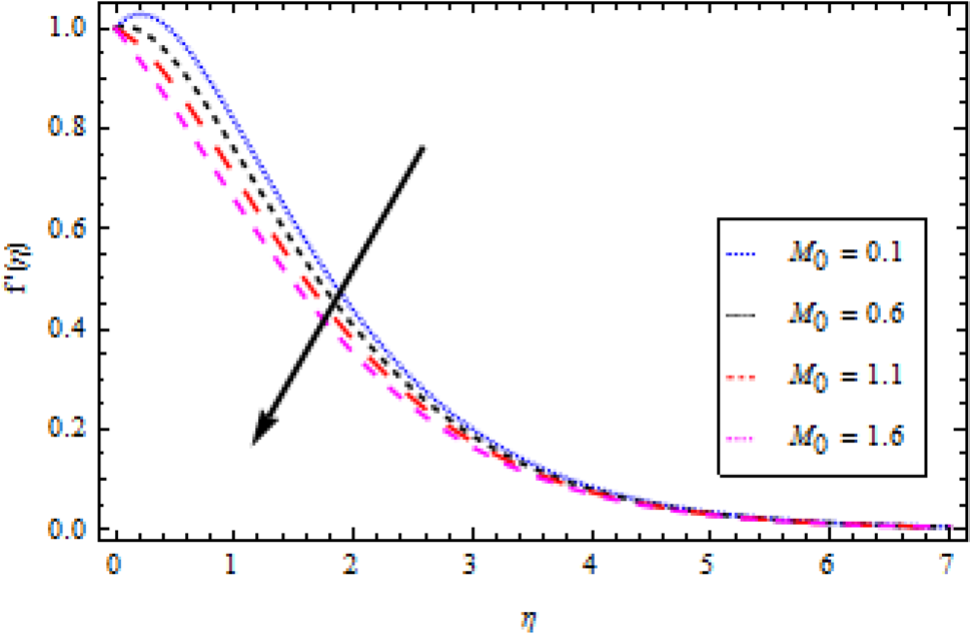
Velocity profile variation with respect to *M*_0_.

**Figure 5:**
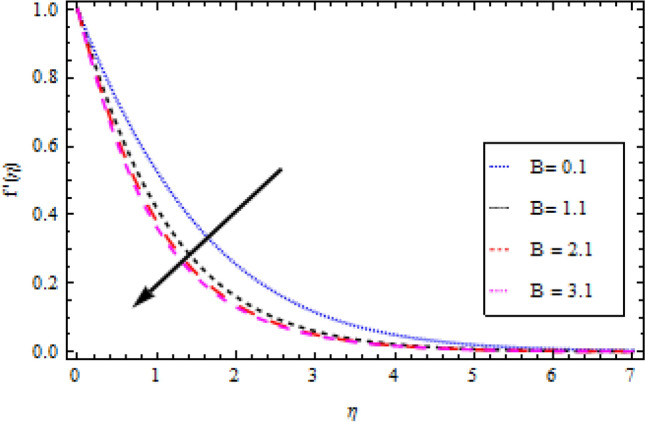
Velocity profile variation with respect to *B.*

In this part, we’ll look at how fluid parameters affect the flow profile. Significant importance is given to fluid parameters such as modified Hartman factor $H$, dimensionaless parameter $B$, fluid parameter $We$, magnetic interaction parameter ${M}_{0}$, heat and mass Biot numbers ${B}_{1}, {B}_{2}$, Brownian motion factor ${N}_{b}$, thermophoretic parameter ${N}_{t}$, Brikman number ${B}_{r}$ and Buoyancy ratio $R$. Figures 2, 3, 4, and 5 show how $We, H, {M}_{0}$ and $B$ affect the velocity profile. The flow profile is greatly fall down when the Williamson parameter $We$ is used. $We$ values that are higher continue to improve the velocity profile. In the presence of multiple dimensionless parameters, the plot of describing the velocity field against $\eta$ for various values of $H$ indicates that the velocity field accelerates with an enlargement in $H$, as shown in Fig. 3. Actually, this the pattern corresponds to the problem’s physical principles in the sense that $H>0$ denotes an assisting flow mechanism on the velocity region. Figure 4 demonstrates the influence of magnetic parameter ${M}_{0}$ over velocity profile. It is noticed that the velocity reduces by increasing ${M}_{0}$. As shown in Fig. 5, as the dimensionless parameter $B$ is increased, the fluid motion decreases.

### Temperature profile

**Figure 6:**
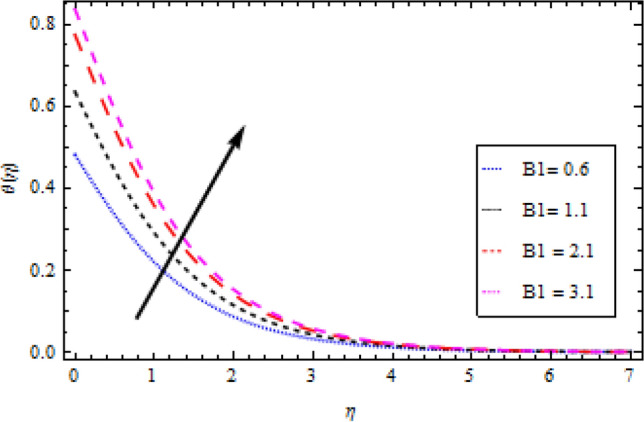
Temperature profile variation with respect to *B*_1_.

**Figure 7:**
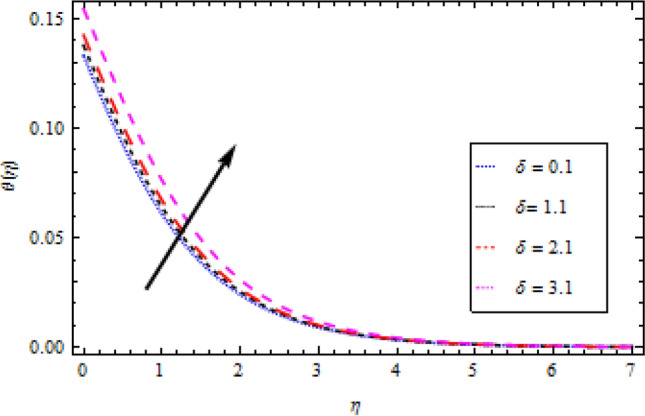
Temperature profile variation with respect to *δ*.

**Figure 8:**
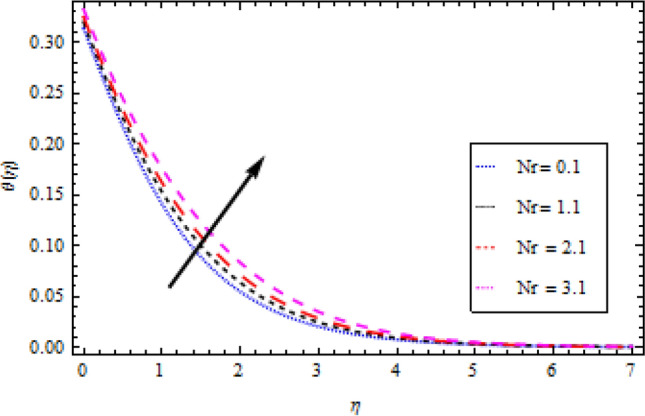
Temperature profile variation with respect to *N*_*r*_.

**Figure 9:**
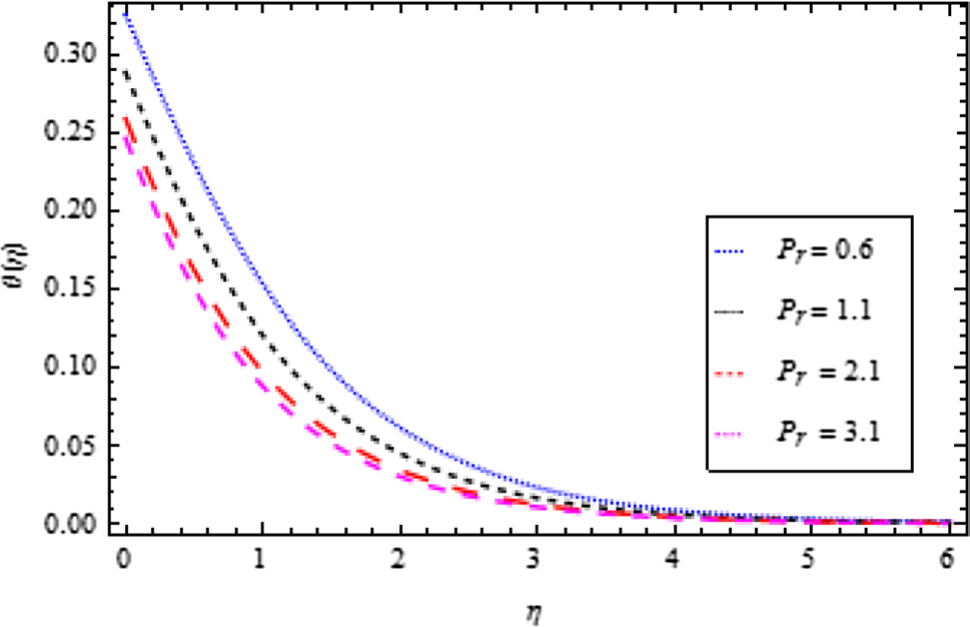
Temperature profile variation with respect to *P*_*r*_.

**Figure 10:**
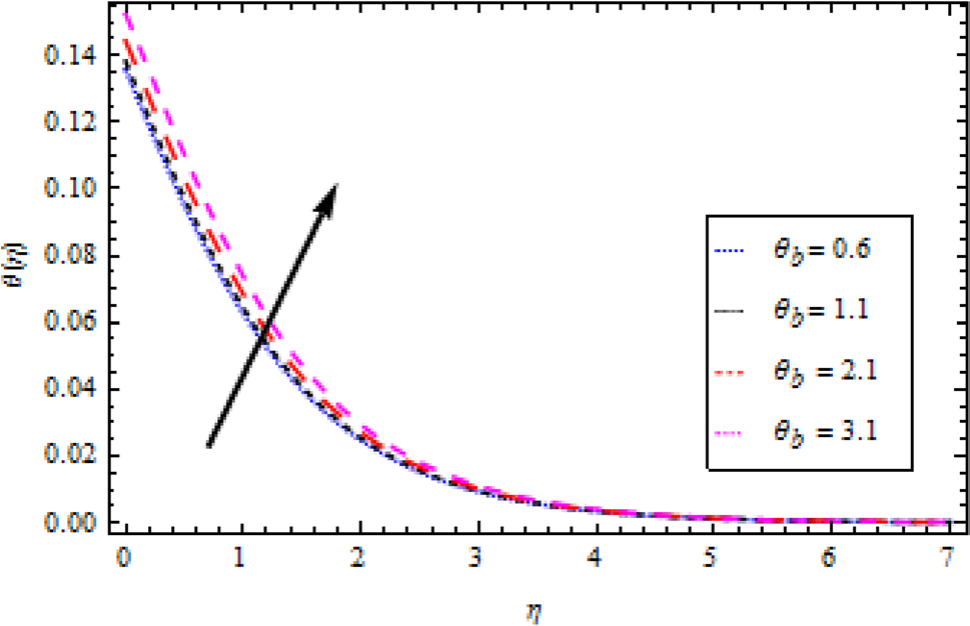
Temperature profile variation with respect to *θ*_*b*_.

The Plots of different fluid factors on the thermal profile are shown in Figs. 6, 7, 8, 9 and 10. Figure 6 depicts the reaction of the surface convection term Biot number ${B}_{1}$ on the thermal sector. As a result of the escalation in ${B}_{1}$, the size of the thermal boundary layer expands and the temperature distribution increases, as seen in the figure. A rise in ${B}_{1}$ strengthens the heat coefficient transport, causing the temperature field to rise. Similarly, with an increase in the magnitude of thermal conductivity term $\delta$, the temperature field improves as demonstrated in Fig. 7. The outcome of the radiation parameter ${N}_{r}$ on the thermal profile is shown in Fig. 8. Due to a durable heat source, increased values of the radiation parameter result in an escalation in temperature. The temperature profile is depicted in Fig. 9 directly influenced by the momentum to mass diffusivity ratio. When the involved parameter ${P}_{r}$ is augmented, a decreasing behavior is observed. A growth in temperature ratio ${\theta }_{b}$ improves the surface temperature as illustrated in Fig. 10.

### Concentration profile

**Figure 11:**
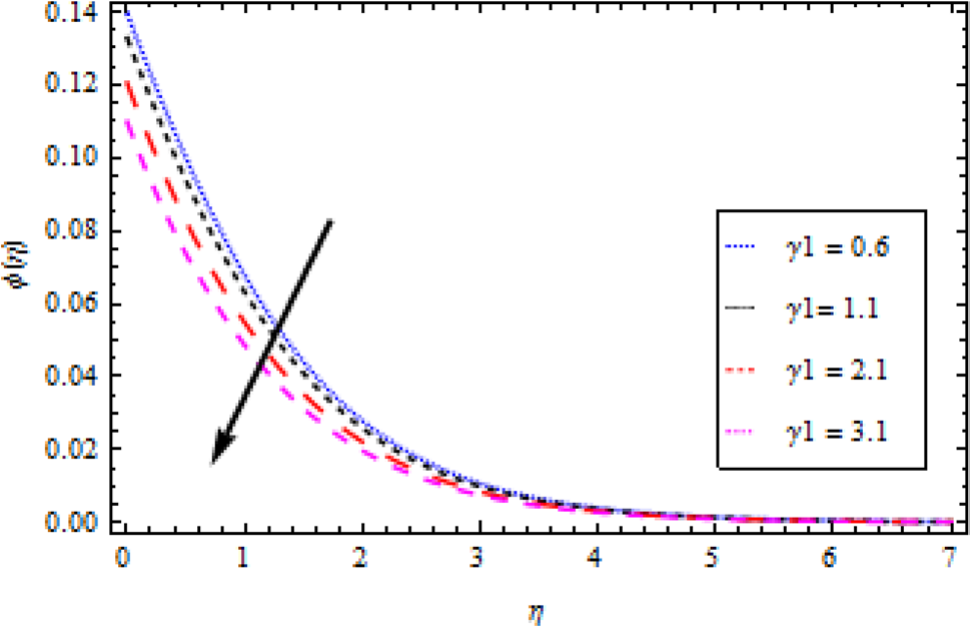
Concentration profile variation with to *γ*_1_.

**Figure 12:**
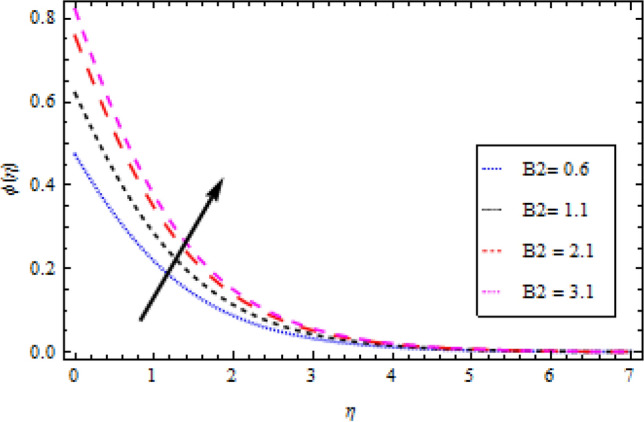
Concentration profile variation with respect to *B*_2_.

**Figure 13:**
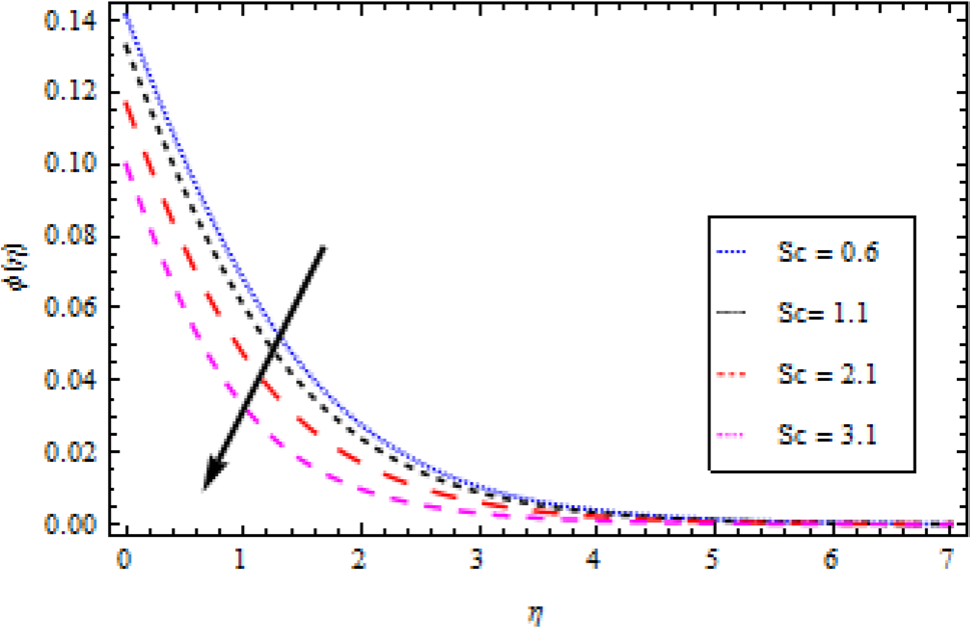
Concentration profile variation with respect to *Sc*.

**Figure 14:**
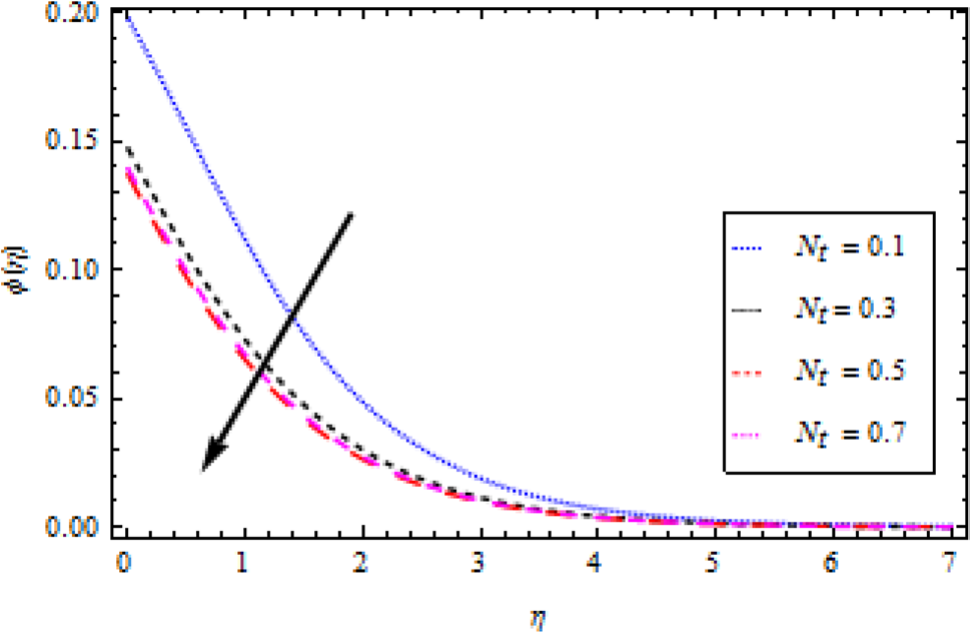
Concentration profile variation with respect to *N*_*t*_.

**Figure 15:**
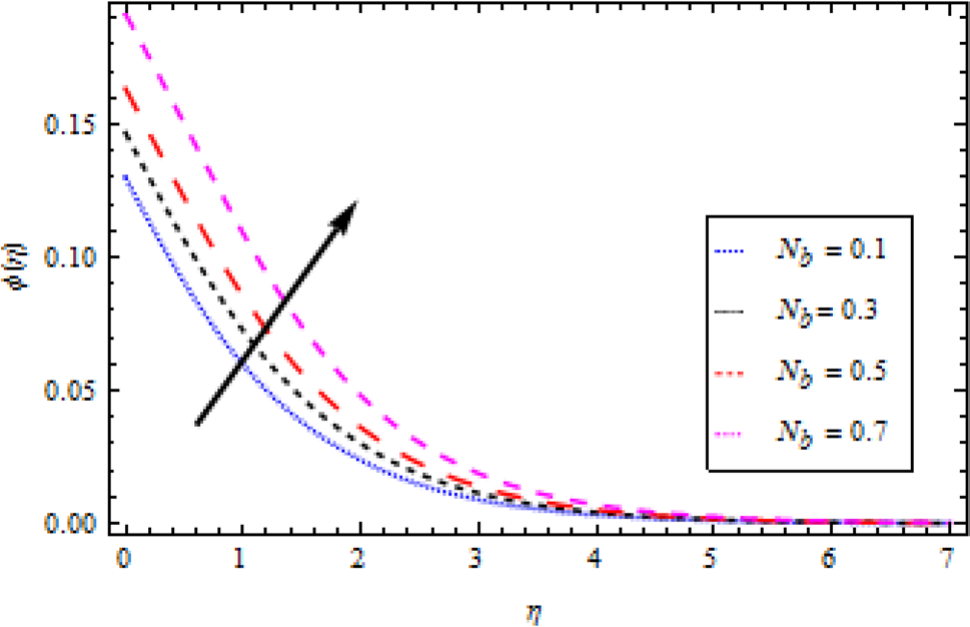
Concentration profile variation with respect to *N*_*b*_.

The graphs in Figs. 11, 12, 13, 14 and 15 show different factors alongside the concentration profile. In Fig. 11 the impression of the chemical reaction factor ${\gamma }_{1}$ on the concentration field is described. It has been discovered that increasing ${\gamma }_{1}$ causes the concentration boundary layer to shrink, lowering the concentration profile. As shown in Fig. 12, the concentration boundary structure is associated with rising values of the mass Biot number ${B}_{2}$. Figure 13 shows a plot of the concentration profile versus $\eta$ for Schmidt number $Sc$ variation. With an upturn in $Sc$, the concentration area alongside the solutes boundary layer thins out. Similarly, Fig. 14 shows a plot of the concentration profile for thermoporesis parameter ${N}_{t}$ variation. The concentration profile decreases by increasing ${N}_{t}$. The outcome of the Brownian motion ${N}_{b}$ on the concentration profile is plotted in Fig. 15. The figure shows that the concentration boundary layer is associated with increasing values of ${N}_{b}$.

### Entropy generation

**Figure 16:**
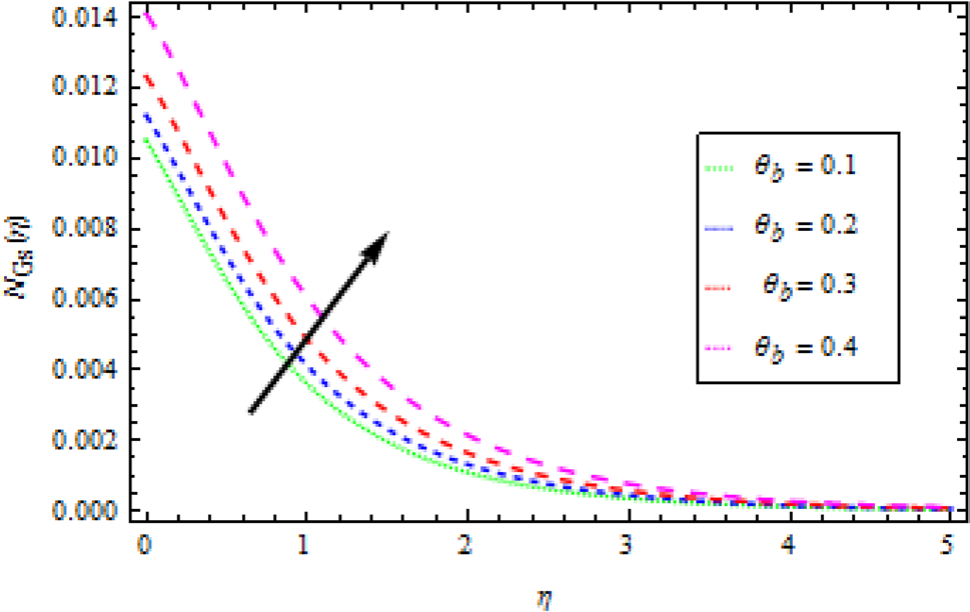
Effects of *θ*_*b*_ on entropy generation.

**Figure 17:**
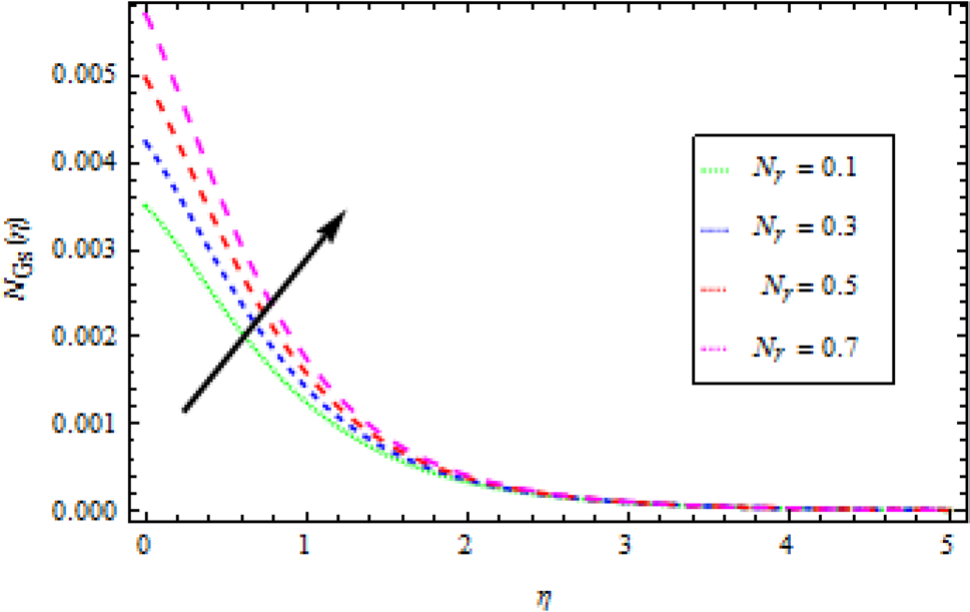
Effects of *N*_*r*_ on entropy generation.

**Figure 18:**
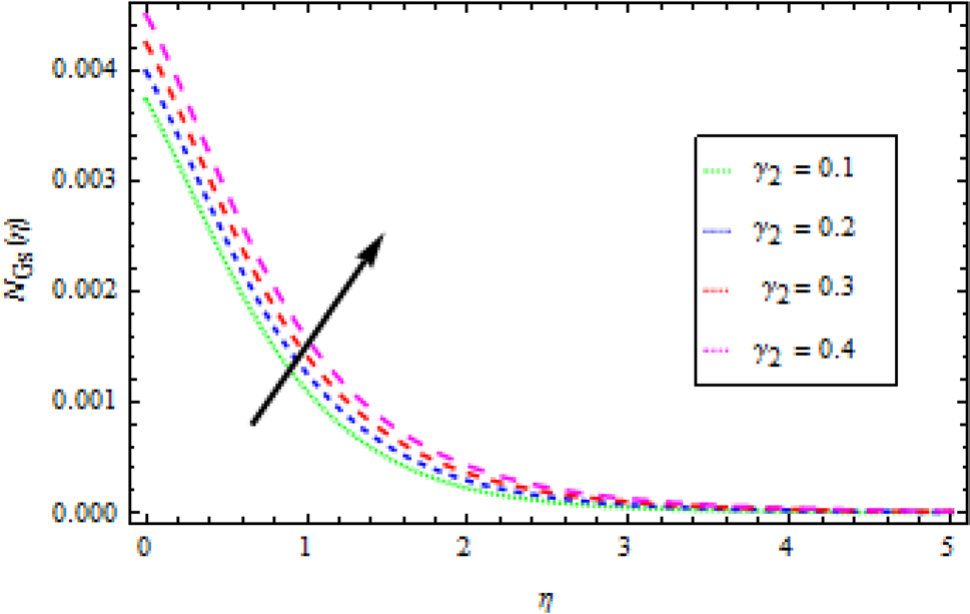
Effects of *γ*_2_ on entropy generation.

Figures 16, 17 and 18 are show graphs of different parameters versus entropy generation. The reaction of entropy generation number ${N}_{Gs}$ to changes in temperature ratio ${\theta }_{b}$ values are depicted in Fig. 16. An uplift in ${\theta }_{b}$ decreases ${N}_{Gs}$. With an increase in ${N}_{r}$, entropy generation ${N}_{Gs}$ also increases, as shown in Fig. 17, which specifies that heat transmission irreversibility dictates entropy generation. It is noticed that increase in radiative parameter increases Bejan number. In Fig. 18, the effect of the diffusion constant ${\gamma }_{2}$ on entropy generation ${N}_{Gs}$ is highlighted. It has been observed that as ${\gamma }_{2}$ increases, ${N}_{Gs}$ increases.

### Bejan number

**Figure 19:**
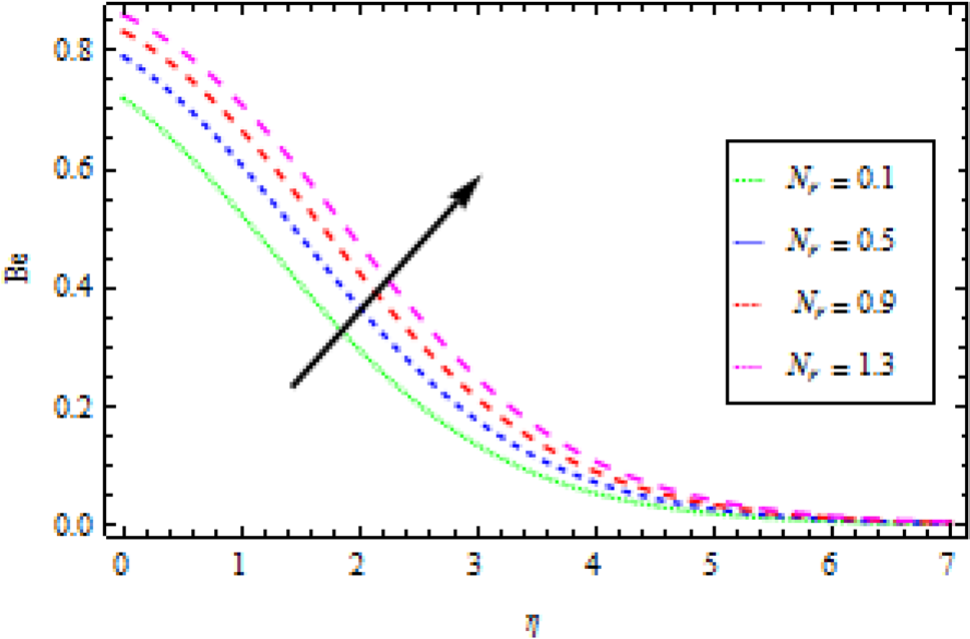
Effects of *N*_*r*_ on Bejan number.

**Figure 20:**
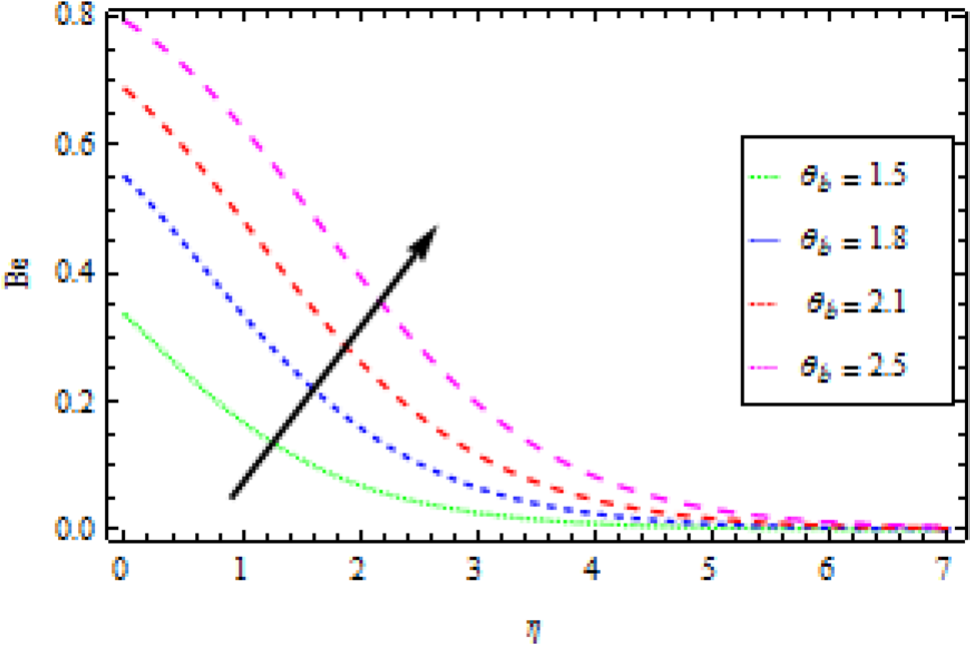
Effects of *θ*_*b*_ on Bejan number.

Figures 19 and 20 show the effects of ${N}_{r}$ and ${\theta }_{b}$ on the Bejan number $Be$, respectively. $Be$ improves with an increase in both ${N}_{r}$ and ${\theta }_{b}$, as seen in these graphs. The assumption is that as the values of ${N}_{r}$ and ${\theta }_{b}$ rise, the entropy generated by heat and mass transfer outnumbers that are produced by frictional heating.

## Conclusion

Entropy production in a reactive Williamson nanofluid MHD movement over a vertical Riga plate has been studied using an analytical solution. The effects of nonlinear thermal radiation, thermophoresis, Brownian motion and varying thermal conductivity are all included in the model. The parametric effects of emerging terms on non-dimensional quantities are graphically represented and discussed as a result. According to the report:

- The velocity profile rises as the fluid parameter $We$, magnetic parameter ${M}_{0}$ and dimensionless number $B$ are increased. As the velocity profile decreases, the modified Hartman number $H$ rises.
- With the increase in surface convection term (Biot number) ${B}_{1}$, thermal conductivity $\delta$, radiation term ${N}_{r}$ and temperature ratio ${\theta }_{b}$, the thermal field expands. While the thermal field shrink with incremental values of Prandtl number.
- By rising the values of mass Biot number ${B}_{2}$ and Brownian motion factor ${N}_{b}$, the concentration profile rises. However, for raising the values of chemical reaction ${\gamma }_{1}$, Schmidt number $Sc$ and thermoporesis parameter ${N}_{t}$, the opposite behaviour was observed.
- With the increase in radiation term ${N}_{r}$ and temperature ratio parameter ${\theta }_{b}$, the entropy generation is high, while opposite behaviour was noticed when diffusion constant term ${\gamma }_{2}$ increased.
- Bejan number rises as the radiation term ${N}_{r}$ and temperature ratio ${\theta }_{b}$ rise.
